# Barriers to International Telemedicine Conferencing: A Survey of the National University Hospital Council of Japan

**DOI:** 10.1089/tmj.2021.0046

**Published:** 2022-03-10

**Authors:** Kuriko Kudo, Noriko Isobe, Shintaro Ueda, Shunta Tomimatsu, Tomohiko Moriyama, Shuji Shimizu

**Affiliations:** ^1^ Telemedicine Development Center of Asia, International Medical Department, Kyushu University Hospital, Fukuoka, Japan.; ^2^ Department of Neurology, Graduate School of Medical Sciences, Kyushu University, Fukuoka, Japan.

**Keywords:** telemedicine, internationalization, national survey, Japan, university hospital

## Abstract

**Introduction:**

Telemedicine conferencing is expected to become commonly used internationally. However, national reports on internationally related telemedicine are limited, and related activities and challenges in each country are unclear. In this study, we aimed to clarify the current status and barriers to international telemedicine conferencing in Japan.

**Methods:**

The questionnaire was sent to the Internationalization Project Team (I-PT) representatives in all 43 Japanese National University Hospitals. The total of 167 assigned staff comprised 86 medical staff in charge of internationalization (MI) and 81 technical staff in telemedicine (TT).

**Results:**

The response rate was 93% (40/43 universities) from 88 staff (44 MI and 44 TT). Most respondents (75%) stated that they had not been active in international telemedicine conferencing during the past 3 years, although a videoconferencing system was installed in 93% of universities. A total of 65% respondents felt that barriers to promoting telemedicine and conferencing existed. Most (43%) respondents reported staff shortage as the most serious barrier overall. Five TT (19%) felt that the most serious barrier was difficulty with English communication, although no MI selected this as a barrier. More MI than TT felt that technical issues were the most serious barrier (MI: 4/29, TT: 1/27).

**Conclusions:**

International telemedicine conferencing was found to be insufficiently active in I-PT of Japan, although the installed equipment and technical expertise of TT seemed adequate. This indicates that merely assigning MI and TT to an I-PT is not enough and that improved cooperation between both MI and TT at each university hospital is needed. Establishment of a structured international telemedicine center in each university hospital is to be suggested to accelerate the activities in Japan.

## Introduction

With a view toward the Tokyo Olympic Games, which have been postponed until 2021, Japan is now in a crucial stage of internationalization.^1,2^ Communication in a common language such as English has been promoted in various situations, including medical facilities that accept patients from foreign countries.^3–5^ The level of medical care in Japan should be globalized to accept foreign patients. At the same time, the coronavirus disease 2019 (COVID-19) pandemic has greatly influenced human activities with substantial, speedy changes in the style of local and international conferences, from face-to-face meetings to videoconferencing (VC).^6,7^ Telemedicine conferencing, which connects international hospitals via VC, enables health care professionals to consult with other health experts around the world, with no travel costs and greatly reduced time requirements.

For example, by attending international case conferences, knowledge and skills, such as how to manage rare diseases and how to control emerging infectious diseases such as COVID-19, can be immediately and safely acquired. In addition, live demonstrations make it possible to view advanced surgical techniques conducted by world-leading experts. Therefore, these telecommunication tools are expected to continue to be more commonly used by health care professionals in the settings of medical education and internationalization. Hoxha et al.^8^ reported the contribution of tele-educational programs in rebuilding the medical system in Kosovo after a decade of war had destroyed the region's health and education systems. Those authors reported activities, including hosting remote lectures and telesurgeries, from 20 countries. Ho et al.^9^ reported the usefulness of case teleconferences among leading hospitals in the Asia Pacific region for upgrading treatment with gastrointestinal endoscopy. Shimizu et al.^10^ reported the effectiveness of live demonstrations given by worldwide experts from their own hospitals, which avoided the time and costs of travel to the transmitting hospitals, and the risks of performing in an unfamiliar environment, such as with unfamiliar medical equipment and staff. National surveys have also been conducted of telemedicine in domestic telehealth expansion^11,12^ and medical school curricula.^13,14^ However, there are a limited number of research reports specifically regarding internationally related telemedicine activities. Therefore, the level of such activities, as well as the challenges in each country, has been unclear.

Under the leadership of the National University Hospital Council of Japan, an Internationalization Project Team (I-PT) was established in 2012.^15^ One aim of the I-PT was to strengthen cooperation with overseas medical institutions through the development and use of information and communications technology (ICT), to establish a world-leading remote medical educational network. Toward this aim, all 43 Japanese national university hospitals assigned staff to one of two roles: (1) medical staff in charge of internationalization (MI) and (2) technical staff in telemedicine (TT).

Although there have been reports on the adoption of international telemedicine conferencing in Japan,^16,17^ few reports exist that summarize activities in Japan overall. Therefore, the activities and challenges in international telemedicine conferencing for Japanese hospitals remain unclear. In this study, we aimed to clarify the current status and barriers to international telemedicine conferencing in the National University Hospital Council of Japan, on the basis of past I-PT activities.

## Methods

Participants in this study were I-PT members in all 43 Japanese national university hospitals. In total, 167 staff were included: 86 MI and 81 TT. We developed a questionnaire regarding characteristics, activities, technical resources, and barriers to international telemedicine conferencing at the hospitals. The questionnaire was created using Google Forms (Google LLC, Mountain View, CA, USA) and was sent via e-mail separately to MI and TT in September 2019. Each university hospital was requested to provide at least one completed questionnaire from both an MI and a TT. The MI questionnaire consisted of 28 questions and the TT questionnaire comprised 43 questions in total. Excerpted questions are shown in *Table 1*. Ethical approval was not required for this research.

**Table 1.** Questionnaire Items

CATEGORY	QUESTIONS	TARGET
Characteristics	1. Please select the response that best describes your job category: *(For MI) Faculty professor (MD)/Faculty professor (not MD)/MD (not faculty professor)/Other* *(For TT) Faculty professor (MD)/Faculty professor (not MD)/MD (not faculty professor)/Administrative staff/IT staff/Other* 2. Percentage of time dedicated to telemedicine: *0–20%/21–40%/41–60%/61–80%/81–100%*	MI, TT
Activities	1. Have you conducted international telemedicine conferencing at your university hospital in the past 3 years? *Yes/No/Not sure* 1–1. (If yes) Please share details: *Free-text response*	MI, TT
Technical resources	1. Are there any VC systems that can be used for telemedicine education at your university hospital? *Yes/No* 2. Please select the response that best describes your level of experience with the following: 2-1. Operating a personal computer 2-2. Operating audiovisual equipment 2-3. Setting up a VC system 2-4. Operating a VC system 2-5. Setting up network devices 2-6. Network connectivity testing 2-7. English communication *Very experienced/Experienced/Neutral/Less experienced/Inexperienced*	TT
Barriers	1. Do you think there are any barriers to promote international telemedicine conferencing at your university hospital? *Yes/No/Not sure* 1–1. (If yes) Among the items below, which is the most serious barrier? *Shortage of medical staff/Shortage of engineers/Not sure how to start/Insufficient support from hospital/Technical issues/Difficulty with collaboration within the hospital/English communication/Policy issues/Difficult working after hours and on holidays/Other*	MI, TT

IT, information technology; MD, medical doctor; MI, medical staff in charge of internationalization; TT, technical staff of telemedicine; VC, videoconferencing.

## Results

### Demographic Features

The survey response rate was 93% (40/43 university hospitals). A total of 88 staff (44 MI and 44 TT) completed the questionnaire. Nearly all (98%, 43/44) MI were faculty professors and medical doctors (MDs). TT included faculty professors (not MDs) (41%, 18/44), information technology (IT) staff (21%, 9/44), faculty professors (MDs) (18%, 8/44), and administrative staff (18%, 8/44). Nearly all respondents (MI: 96%, TT: 93%) reported that less than 20% of their time was dedicated to telemedicine (*Table 2*).

**Table 2.** Characteristics of Survey Respondents

	MI	*N* = 44, *n* (%)	TT	*N* = 44, *n* (%)
Occupation	Faculty professor (MD)	43 (98)	Faculty professor (MD)	8 (18)
	Faculty professor (not MD)	1 (2)	Faculty professor (not MD)	18 (41)
	MD (not faculty professor)	0	MD (not faculty professor)	1 (2)
	Other	0	Administrative staff	8 (18)
			IT staff	9 (21)
Percentage of effort dedicated to telemedicine	0–20%	42 (96)	0–20%	41 (93)
	21–40%	1 (2)	21–40%	1 (2)
	61–80%	1 (2)	61–80%	0
	81–100%	0	81–100%	2 (5)

### Activities

Most respondents (75%, 66/88) stated they had not implemented any international programs or did not know whether a program had been implemented in their institution during the past 3 years. The remaining 25% of respondents (22/88), representing 13 university hospitals (33%, 13/40), had implemented programs in a variety of medical subspecialties such as surgery, neurology, ophthalmology, infectious diseases, and public health. Regarding counterpart countries, most university hospitals (77%, 10/13) had conducted telemedicine conferencing activities with other Asian countries.

### Technical Resources

**Fig. 1.**
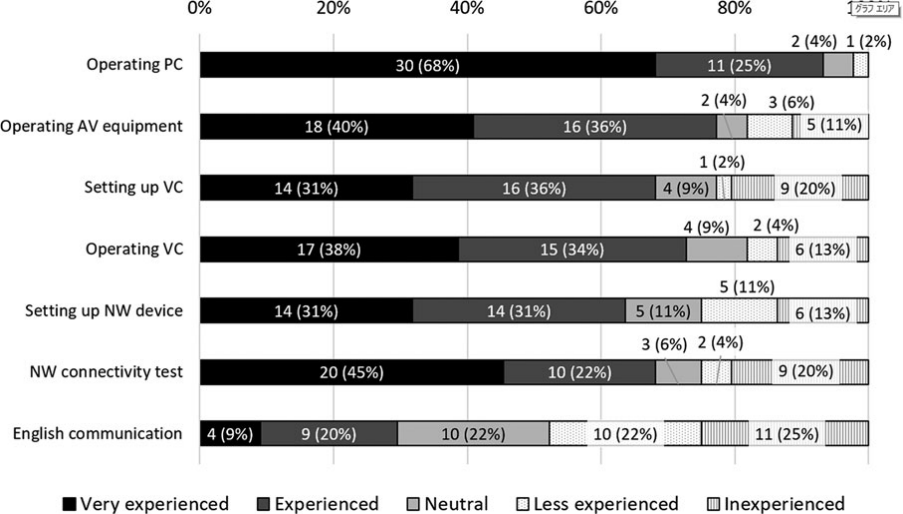
Experience of technical staff in telemedicine (*N* = 44). AV, audiovisual; NW, network; PC, personal computer; VC, videoconferencing.

VC systems were installed in 93% (37/40) of university hospitals. As for technical skills, 93% of respondents (41/44) had experience in computer operations, audiovisual equipment operation (77%, 34/44), and VC system operation (73%, 32/44); more than half (64%, 28/44) had experience with network device settings. However, only 30% (13/44) reported a high level of experience with communication in English (*Fig. 1*).

### Barriers to International Telemedicine Conferencing

Most respondents (65%, 57/88) stated that barriers existed to promoting international telemedicine conferencing at their university hospitals. The most serious barrier was a shortage of staff such as doctors and engineers (43%, 24/56), followed by not being sure how to begin (14%, 8/56), technical issues (9%, 5/56), and communication in English (9%, 5/56). No respondents reported policy issues as the most serious barrier (*Table 3*). The five respondents who selected other barriers reported difficulties in providing acknowledgment to counterparts (2), no incentives for technical staff (1), laws and systems regarding personal information (1), and problems with human resources (1).

**Table 3.** Most Serious Barriers to Promoting International Telemedicine Conferencing

	OVERALL	*N* = 56, *n* (%)
1	Staff shortage	24 (43)
2	Not sure how to start	8 (14)
3	Technical issues	5 (9)
	English communication	5 (9)
4	Difficulty working after hours and on holidays	4 (7)
5	Difficult collaboration within the hospital	3 (5)
6	Insufficient support from the hospital	2 (4)
7	Other	5 (9)

When comparing the presence of activities, staff shortage was the most selected in both groups (Activity [−]: 49%, Activity [+]: 32%). Here, activities (+) and (−) were grouped based on if they had carried out international telemedicine conferencing in the past 3 years, respectively. However, no one in the activity (+) group selected “not sure how to start,” which was the second leading barrier in the activity (−) group (22%, 8/37). The response “technical issues” was the second-most commonly selected (21%, 4/19) in the activity (+) group; only one (1/37) respondent in the activity (−) group selected this as the most serious barrier (*Table 4*).

**Table 4.** Most Serious Barriers to Promoting International Telemedicine Conferencing, by Activity Status

	ACTIVITY (−)	*N* = 37, *n* (%)	ACTIVITY (+)	*N* = 19, *n* (%)
1	Staff shortage	18 (49)	Staff shortage	6 (32)
2	Not sure how to start	8 (22)	Technical issues	4 (21)
3	English communication	3 (8)	English communication	2 (11)
	Difficult collaboration within the hospital	3 (8)	Difficulty working after hours and on holidays	2 (11)

When comparing MI and TT, both reported staff shortages as the main barrier (MI: 45%, TT: 41%); however, more MI reported not being sure how to begin (MI: 6/29, TT: 2/27) and technical issues (MI: 4/29, TT: 1/27) as the barriers. In contrast, 19% (5/27) of TT stated that English communication was the most serious problem, whereas no MI reported this. Difficulty working after hours and on holidays was the third-most common barrier selected by TT (3/27), but only one MI selected this response (*Table 5*).

**Table 5.** Most Serious Barriers to Promoting International Telemedicine Conferencing, by Staff Roles

	MI	*N* = 29, *n* (%)	TT	*N* = 27, *n* (%)
1	Staff shortage	13 (45)	Staff shortage	11 (41)
2	Not sure how to start	6 (21)	English communication	5 (19)
3	Technical issues	4 (14)	Difficulty working after hours and on holidays	3 (11)

## Discussion

In this study, we revealed that most respondents (75%) in 68% (27/40) of universities were not actively engaged in international telemedicine conferencing, despite this being set as an important goal by the National University Hospital Council of Japan. The results demonstrated the efficiency of technical resources, such as VC systems installed in nearly all institutions. Most TT only had experience in basic skills required to implement telemedicine conferencing, which most (65%) felt was a barrier to promoting international telemedicine conferencing at their hospitals, and which seemed to be the cause of the low level of related activities.

### Staff Shortages

The shortage of staff, such as doctors and engineers, was selected as the most serious barrier to international telemedicine conferencing by most survey respondents. MI and TT are in charge of such activities at university hospitals; however, the average reported percentage of time dedicated to telemedicine and telecommunication was less than 20%. MI and TT mainly work in other roles, such as in clinical care and medical research among MI, and management of electronic health record systems and administrative procedures among TT. As a result, there are few human resources available for telemedicine activities.

### Unsure how to Begin

Although not selected by any respondents in the activity (+) group, being unsure how to begin was the second-most common response selected by the activity (−) group. Therefore, this barrier must be resolved to engage in telemedicine activities. Conducting seminars for beginners and inviting them to participate in pilot programs may be one solution. Inviting beginners to participate in existing training programs will be also effective, such as Project Echo, which provides a variety of training programs in telemedicine.^18,19^ Although these training programs are recommended to be included in medical school curricula, this remains uncommon. A 2016 national survey of all 19 medical schools in Australia revealed that the curricula do not include e-health programs.^13^ In France, telemedicine education and training are limited in medical schools, even though 90% of deans agree with its importance.^14^

### Technical Issues

Technical issues were the second-most commonly reported barrier in the activity (+) group. Recently, the usability of VC has improved and it has become relatively easier than in the past.^20^ Therefore, many users are now able to set up and control VC on their own.^21,22^ However, technical issues can easily occur in international telemedicine conferencing, depending on the available networks and equipment.^9,23,24^ This issue can be resolved with appropriate technical support. Tomimatsu et al.^25^ reported that the presence of technical staff prevented technical issues in large multiparty medical videoconferences. The responses of TT indicated no problems with respect to equipment and technical experience; this must be the reason why only one (4%) TT selected this item as the most serious barrier. However, more MI (4, 14%) chose this item as the most serious barrier, which shows that there is a distinction between MI and TT in terms of technical issues.

### Difficulty with Communication in English

English communication was the second-most commonly reported barrier in the TT group, although no MI considered this a problem. Many physicians who belong to university hospitals often partake in conversations in English, for example, at an international congress; however, most IT and administrative staff have no such experience. When establishing VC connections, technical communication between technical staff and overseas partners is mainly conducted in English. Therefore, if TT can receive support with translation, technical setup and implementation of VC should work more smoothly. English communication skills have become an important evaluation criterion for technical personnel. If needed, providing language support from other staff in the hospital is also recommended.

### Difficulty Working After Hours and on Holidays

Working after hours or on weekends was the third-most serious barrier in the TT group. When working with people in other countries, working outside of normal hours may be required owing to time differences. However, most MI did not report this as the most serious barrier. One reason is that technical staff must work longer than participants during teleconferences as they perform activities such as setup and connectivity testing before a conference, and dismantlement of equipment after a conference. Another reason is that working hours among Japanese physicians are longer than those in other occupations, so overtime may not be regarded as a barrier for MI.^26^

### Difficulty with Collaboration within Hospitals

This was the third-most commonly reported barrier in the activity (−) group, even though no one selected this in the activity (+) group. To implement international telemedicine conferencing, collaboration between many different staff participants is needed, such as physicians, nurses, technical staff, administrators, and international coordinators. Therefore, it is important to decide the roles of each of these diverse staff members, which might become a barrier. Furthermore, it is difficult to start something new, especially in a large organization such as a university hospital.

Our results concerning barriers to international telemedicine conferencing showed that the current system, which only assigns MI and TT to the I-PT, is inadequate to promote international telemedicine conferencing in Japanese university hospitals. The differences in barriers considered most problematic among MI and TT indicated a lack of collaboration between MI and TT. Therefore, strengthening cooperation between both groups is needed, not by establishing two different roles but rather by establishing an “international telemedicine center” at each university hospital and assigning both MI and TT to be involved in the related activities. In this way, many of the reported barriers could be resolved. For example, the barrier of technical issues among MI could be resolved by providing technical support from TT, and the English language and working hour barriers could be addressed by obtaining translation and incentives from MI and university hospitals, respectively. Collaboration within each university hospital can be improved if the international telemedicine center plays a central role in coordinating the activities of a variety of hospital staff.

As an example, researchers at the Mayo Clinic in the United States reported difficulties in sharing information among telemedicine projects, as these projects had been developed within individual departments. The Mayo Clinic established a center for telemedicine as a dedicated department, and provider satisfaction was increased with fewer technical issues.^27^ Although their telemedicine center was not intended for international remote educational purposes and not exactly the same as ours, the current situation is similar in many Japanese national university hospitals as there are no dedicated departments and staff for international telemedicine conferencing. Therefore, such projects may be carried out in isolation and not made transparent to all departments and staff.

This study has some limitations. Although the study was conducted regarding the I-PT, the responsibility of the representatives differed by institution. Furthermore, respondents' dedication to telemedicine was less than 20% in nearly all cases; therefore, this study was based on limited information. However, the I-PT plays a unique role in promoting international telemedicine conferencing at national university hospitals, so summarizing the current status among I-PT members should be the first step in understanding the situation of international telemedicine conferences in Japan. At least one person from each university hospital was requested to complete the survey, so the results may include individual bias of respondents. Another limitation is that the questionnaire only provided simple response options. More thorough analysis should be performed using qualitative study methods. Moreover, statistical analysis is needed. The status regarding international telemedicine conferencing before the COVID-19 pandemic has been summarized in this article. However, the situation post-COVID-19 would be different as staff in medical institutions have been forced to shift to greater use of online communication.^28,29^

Japan has been said to be one of the slowest countries to adopt internationalization. Saiki et al.^30^ analyzed the current status of internationalization in Japanese medical education and pointed out the extremely limited output toward foreign countries, although input, such as “translationalism” with an open mindset for international trends, has been developing in Japan. Hence, greater mutual collaboration with foreign countries is suggested for Japanese medical staff and students. Health care professionals worldwide are now in serious need of communication with other countries to exchange information on controlling COVID-19. ICT allows us to communicate locally and globally with no risk of infection. In the future, more medical institutions will communicate using ICT, nationally and internationally, which will contribute to internationalization of health care and medical professionals in Japan.

## Conclusion

In this study, we clarified the current status and barriers to international telemedicine conferencing in Japan, using questionnaires administered to MI and TT of the I-PT, National University Hospital Council of Japan. International telemedicine conferencing is not sufficiently active in Japan, even though the installed equipment and technical expertise of TT are adequate. Barriers to promoting such activities were clarified, such as staff shortages, being unsure how to begin, technical issues, and difficulty with communication in English. Furthermore, a lack of collaboration between MI and TT was revealed. Establishment of an international telemedicine center is suggested, to address insufficient collaboration between these groups and to promote international telemedicine conferencing activities at national university hospitals in Japan.
